# Association between number of teeth and Alzheimer’s disease using the National Database of Health Insurance Claims and Specific Health Checkups of Japan

**DOI:** 10.1371/journal.pone.0251056

**Published:** 2021-04-30

**Authors:** Midori Tsuneishi, Tatsuo Yamamoto, Takeyuki Yamaguchi, Tsuyoshi Kodama, Tamotsu Sato

**Affiliations:** 1 Japan Dental Association Research Institute, Tokyo, Japan; 2 Department of Dental Sociology, Kanagawa Dental University, Yokosuka, Japan; 3 Japan Dental Association, Tokyo, Japan; Ehime University Graduate School of Medicine, JAPAN

## Abstract

Associations of numbers of teeth present and of missing teeth with Alzheimer’s disease were cross-sectionally analyzed using the National Database of Health Insurance Claims and Specific Health Checkups of Japan. Dental care claims data of patients aged 60 years or older diagnosed with periodontitis (n = 4,009,345) or missing teeth (n = 662,182) were used to obtain information about the numbers of teeth present and of missing teeth, respectively, and they were combined with medical care claims data including the diagnosis of Alzheimer’s disease. Numbers of teeth present and of missing teeth excluding third molars were calculated using the dental formula in the claims for periodontitis and missing teeth, respectively, and categorized into three groups each. Percentages of subjects treated for Alzheimer’s disease with 20–28, 10–19, and 1–9 teeth present were 1.95%, 3.87%, and 6.86%, respectively, in patients diagnosed as having periodontitis, and those treated for Alzheimer’s disease with 1–13, 14–27, and 28 missing teeth were 2.67%, 5.51%, and 8.70%, respectively, in patients diagnosed as having missing teeth. Logistic regression models using treatment for Alzheimer’s disease as an outcome variable and adjusting for age and sex showed that odds ratios (95% confidence intervals) for patients with 10–19 and 1–9 teeth (reference: 20–28 teeth) were 1.11 (1.10–1.13) and 1.34 (1.32–1.37), respectively, (*p*<0.001), in patients diagnosed as having periodontitis, and odds ratios (95% confidence intervals) for patients with 14–27 missing teeth and 28 missing teeth (reference: 1–13 missing teeth) were 1.40 (1.36–1.44) and 1.81 (1.74–1.89), respectively, (*p*<0.001), in patients diagnosed as having missing teeth. In conclusion, the results of the present study using Japanese dental claims data showed that older people visiting dental offices with fewer teeth present and a greater number of missing teeth are more likely to have Alzheimer’s disease.

## Introduction

Dementia in aging populations is one of the largest health and economic issues not only worldwide, but also in Japan. It is expected that the number of people affected by dementia will increase to 66 million by 2030 and 131 million worldwide by 2050 [1]. Dementia affects not only individuals with the condition, but also their relatives and other caretakers, and the annual global cost of dementia including family, social, and medical care is estimated to be US $818 billion [1]. In Japan, the proportion of people aged ≥65 years was about 28% in 2019, and the country is now regarded as a super-aging society. The prevalence of dementia according to 5-year age strata between 65 and 99 years was 5.8%–77.7% in Japan [2], and the prevalence has recently increased [3]. As the number of older people and the prevalence of dementia increase, dental teams will become more likely to encounter oral health problems in people with dementia.

Many studies have shown relationships between cognitive impairment and poor oral health [4]. Cross-sectional studies showed an association between cognitive impairment and poor oral health, including missing teeth [5–8] and major causes of tooth loss, such as dental caries [5, 6, 9] and periodontal disease [6, 10]. Cohort studies suggested that cognitive decline results in poor oral health such as dental plaque accumulation [11], periodontal disease progression [11], and tooth loss [12]. In contrast, case-control studies proposed that a history of tooth loss in early life was a risk factor for dementia [13, 14]. Moreover, cohort studies suggested that tooth loss is a risk factor for cognitive decline [15–19]. These results imply that older patients visiting dental offices with missing teeth might suffer from cognitive impairment. However, little information is available regarding the extent to which older people with cognitive impairment visit dental offices and whether an association between number of teeth present and cognitive impairment exists in patients visiting dental offices.

The National Database of Health Insurance Claims and Specific Health Checkups of Japan (NDB) is a national administrative claims database that covers more than 126 million people and 1.9 billion electronic claims annually in Japan [20]. This database includes almost all (≥95%) claims data regarding medical and dental treatments and specific health checkups and provides a complete picture of the real-world clinical situation in Japan. We have reported associations between number of teeth and dental and medical care expenditures, and number of teeth and medical visits due to aspiration pneumonia using this database [21–23]. The purpose of this study was to clarify the association between number of teeth and Alzheimer’s disease using the NDB. In addition, the prevalence of Alzheimer’s disease in patients visiting dental offices was also evaluated.

## Materials and methods

### Data source

We conducted a cross-sectional study using NDB data on medical and dental claims in April 2017. The NDB, which was developed by the Japanese Ministry of Health, Labour and Welfare, covers almost all patients who receive dental and medical care services under the universal health insurance system [24]. The claims data include clinical and procedural information, such as the patient identification number, sex, age, procedural codes, and diagnostic codes.

After a review of our study protocol by the NDB expert council, we entered a contract with the Ministry to use a dataset extracted from the NDB for the purpose of the present study. We adhered to the guideline on the use of the NDB, based on which we were obligated to use the dataset only in a pre-specified secure room. Informed consent was not obtained because this study used anonymized claims data. This study was approved by the Ethics Committee of the Japanese Association for Dental Science (September 5, 2018, approval number 010) and conducted in full accordance with the guidelines set forth by the World Medical Association Declaration of Helsinki.

Because NDB data do not include information about the number of teeth present, we calculated the number of teeth present using the dental formula, information about tooth type, and information regarding the diagnosis of periodontitis. Patients who undergo periodontal treatment including supportive periodontal treatment or periodontal maintenance are diagnosed with periodontitis using the dental formula of all teeth present. In the NDB, information for “one tooth” for the diagnosis of periodontitis was input using six-digit numbers including information about tooth type. For example, the value for 2 teeth is a 12-digit number, and that for 28 teeth is a 168-digit number. Using the data of the dental formula, the number of digits was counted to estimate the number of teeth present. The validity of the calculated number of teeth present has been confirmed in our previous study by comparing the data with those from the national survey in Japan [25]. In addition, because subjects with periodontitis do not include edentulous patients, i.e., patients without teeth, we used the dental formula to calculate the number of missing teeth for prosthodontic treatment, including bridges and partial and full dentures [22].

Data of patients aged 60 years or older who were diagnosed as having periodontitis (diagnosis code: 5234009, n = 4,009,345) and missing teeth (diagnosis code: 5250001, n = 662,182) were identified and combined with medical care claims data of patients diagnosed with Alzheimer’s disease (diagnosis codes: 8842548 (presenile dementia of the Alzheimer type), 8842549 (dementia of the Alzheimer type), 8842550 (atypical dementia of the Alzheimer type), and 8842551 (senile dementia of the Alzheimer type)) using the identification number generated from the insurance identification number. For patients who had two or more data points regarding periodontitis, only data containing the highest number of teeth present were included in the present analysis. For patients who had two or more data points regarding missing teeth, only data containing the highest number of missing teeth were included in the present analysis.

### Statistical analyses

Numbers of teeth present and of missing teeth were calculated using the dental formula, excluding third molars, of periodontitis and of missing teeth in the NDB data, respectively. Because the number of teeth present was not normally distributed, it was categorized into three groups (1–9, 10–19, and 20–28) [22]. Likewise, the number of missing teeth was not normally distributed, and it was categorized into three groups (1–13, 28, and 14–27, assuming patients missing few teeth, complete loss of teeth and others, respectively) [22].

Percentages of subjects treated for Alzheimer’s disease at least once by a medical doctor in each sex, each age group, those with 20–28, 10–19, and 1–9 teeth and those with 1–13, 14–27, and 28 missing teeth were calculated, and comparisons of the prevalence of Alzheimer’s disease were made by sex, age group, numbers of teeth present and of missing teeth using chi-squared tests. Odds ratios (ORs) [95% confidence intervals (95% CI)] for subjects with 10–19 teeth and 1–9 teeth (reference: 20–28 teeth) were calculated using logistic regression models adjusted for age and sex using treatment for Alzheimer’s disease as an outcome variable. Moreover, ORs (95% CI) for subjects with 14–27 missing teeth and 28 missing teeth (reference: 1–13 missing teeth) were calculated using logistic regression models adjusted for age and sex using treatment of Alzheimer’s disease as an outcome variable.

All analyses were performed using Microsoft Excel 2013 (Microsoft Co., Redmond, WA, USA), Microsoft SQL Server 2008 (Microsoft Co.), and IBM SPSS Statistics 24.0 for Windows (SPSS Japan Inc., Tokyo, Japan), with a significance level of 5%.

## Results

Prevalences of Alzheimer’s disease were 3.0% and 3.7% in subjects diagnosed as having periodontitis and missing teeth, respectively (Tables 1 and 2). Associations of Alzheimer’s disease with sex, age group, and number of teeth present in subjects diagnosed as having periodontitis are shown in Table 1. Women, older subjects, and subjects with fewer teeth present had relatively higher percentages of Alzheimer’s disease (*p*<0.001). In particular, 16.4% of subjects aged 85 years or older and 6.9% of subjects with 1–9 teeth present had Alzheimer’s disease.

**Table 1:** Prevalence of Alzheimer’s disease by sex, age group, and number of teeth present in subjects diagnosed as having periodontitis.

		Total subjects		Subjects with Alzheimer’s disease		*p*-value*
		N	%	N	%	
Sex	Male	1,685,921	42.0	34,561	2.05	<0.001
	Female	2,323,424	58.0	86,607	3.73	
Age group (y)	60–64	591,131	14.7	897	0.15	<0.001
	65–69	898,217	22.4	3,114	0.35	
	70–74	823,287	20.5	7,331	0.89	
	75–79	794,723	19.8	17,912	2.25	
	80–84	538,301	13.4	32,311	6.00	
	85–	363,686	9.1	59,603	16.39	
Number of teeth present (excluding third molars)	20–28	2,483,055	61.9	48,360	1.95	<0.001
	10–19	1,066,458	26.6	41,276	3.87	
	1–9	459,832	11.5	31,532	6.86	
Total		4,009,345	100.0	121,168	3.02	

*Chi-squared test.

**Table 2:** Prevalence of Alzheimer’s disease by sex, age group, and number of missing teeth in subjects diagnosed as having missing teeth.

		Total subjects		Subjects with Alzheimer’s disease		*p*-value*
		N	%	N	%	
Sex	Male	296,788	44.8	7,874	2.65	<0.001
	Female	365,394	55.2	16,482	4.51	
Age group (y)	60–64	60,287	9.1	112	0.19	<0.001
	65–69	116,901	17.7	473	0.40	
	70–74	128,126	19.3	1,246	0.97	
	75–79	144,309	21.8	3,528	2.44	
	80–84	118,401	17.9	6,770	5.72	
	85–	94,158	14.2	12,227	12.99	
Number of missing teeth (excluding third molars)	1–13	469,504	70.9	12,549	2.67	<0.001
	14–27	155,293	23.5	8,554	5.51	
	28	37,385	5.6	3,253	8.70	
Total		662,182	100.0	24,356	3.68	

*Chi-squared test.

Associations of Alzheimer’s disease with sex, age group, and number of missing teeth in subjects diagnosed as having missing teeth are shown in Table 2. Women, older subjects, and subjects with more missing teeth had relatively higher percentages of Alzheimer’s disease (*p*<0.001). Notably, 13.0% of subjects aged 85 years or older and 8.7% of subjects with 28 missing teeth had Alzheimer’s disease.

Results of logistic regression analysis for Alzheimer’s disease with sex, age group, and number of teeth present in subjects diagnosed as having periodontitis are shown in Table 3. ORs (95% CIs) for subjects with 10–19 teeth and 1–9 teeth (reference: 20–28 teeth) were 1.11 (1.10–1.13) and 1.34 (1.32–1.37), respectively (*p*<0.001). Very high (14–112) ORs were noted in subjects aged 75 years or older compared with those aged 60–64 years.

**Table 3:** Results of logistic regression analysis for Alzheimer’s disease with sex, age group, and number of teeth present in subjects diagnosed as having periodontitis.

Independent variable		Odds ratio	95% confidence interval		*p*-value
			Lower limit	Upper limit	
Sex	Male	1.00	(Reference)		
	Female	1.60	1.58	1.62	<0.001
Age group (y)	60–64	1.00	(Reference)		
	65–69	2.25	2.09	2.43	<0.001
	70–74	5.72	5.34	6.13	<0.001
	75–79	14.48	13.54	15.49	<0.001
	80–84	39.02	36.51	41.71	<0.001
	85–	112.32	105.10	120.02	<0.001
Number of teeth present (excluding third molars)	20–28	1.00	(Reference)		
	10–19	1.11	1.10	1.13	<0.001
	1–9	1.34	1.32	1.37	<0.001

Dependent variable: having Alzheimer’s disease (yes/no).

Results of logistic regression analysis for Alzheimer’s disease with sex, age group, and number of missing teeth in subjects diagnosed as having missing teeth are shown in Table 4. ORs (95% CIs) for subjects with 14–27 missing teeth and 28 missing teeth (reference: 1–13 missing teeth) were 1.40 (1.36–1.44) and 1.81 (1.74–1.89), respectively (*p*<0.001). Very high (13–67) ORs were noted in subjects aged 75 years or older compared with those aged 60–64 years.

**Table 4:** Results of logistic regression analysis for Alzheimer’s disease with sex, age group, and number of missing teeth in subjects diagnosed as having missing teeth.

Independent variable		Odds ratio	95% confidence interval		*p*-value
			Lower limit	Upper limit	
Sex	Male	1.00	(Reference)		
	Female	1.42	1.38	1.46	<0.001
Age group (y)	60–64	1.00	(Reference)		
	65–69	2.13	1.74	2.62	<0.001
	70–74	5.04	4.16	6.12	<0.001
	75–79	12.51	10.36	15.10	<0.001
	80–84	29.03	24.08	35.01	<0.001
	85–	66.59	55.25	80.25	<0.001
Number of missing teeth (excluding third molars)	1–13	1.00	(Reference)		
	14–27	1.40	1.36	1.44	<0.001
	28	1.81	1.74	1.89	<0.001

Dependent variable: having Alzheimer’s disease (yes/no).

## Discussion

The results of this cross-sectional study using NDB data of about 4.7 million people showed that patients visiting dentists with fewer teeth present and a greater number of missing teeth are more likely to have Alzheimer’s disease after adjusting for sex and age. The results of the present study agree with those from previous studies using data from <10,000 general subjects, which suggested that cognitive impairment is a risk factor for tooth loss and vice versa [5–19]. Because of the nature of NDB data, this study did not adjust for possible confounders other than sex and age. However, the results of the present study using dental claims data clearly showed that older age, female, and fewer teeth were independently associated with having Alzheimer’s disease in patients visiting dental offices. These results provide useful information for both dentists and medical doctors to understand the present situation of dental visits by patients with Alzheimer’s disease because special attention has to be given to patients with the disease at oral health assessment, oral health care planning, dental treatment, and delivery of dental care [26].

Although the mechanisms underlying the association between tooth loss and Alzheimer’s disease are still unclear, several possibilities could be proposed for the association. First, Alzheimer’s disease might reduce cognition and dexterity in oral hygiene measures, and the poor oral hygiene might increase the risk of periodontal disease and dental caries, and result in tooth loss [11, 12]. Second, tooth loss might increase the risk of Alzheimer’s disease through reduction of mastication [13–19]. Degenerative changes in the cognitive domains of the brain occur due to decreased mastication-induced stimuli to the brain [27]. Moreover, as chewing ability decreases, intake of food such as raw vegetables decreases, and this is expected to cause nutritional deficiencies (e.g., vitamins) [28]. Insufficient intake of nutrients such as vitamins constitutes a risk factor for dementia onset, and thus, the route involving these nutrients is also a possibility [29]. In addition, there might be factors including educational attainment, income, smoking, and diabetes mellitus, which increase the risk of both tooth loss and Alzheimer’s disease [30, 31].

Due to the difference in the cut-off values of the categorization between the number of teeth present in subjects diagnosed as having periodontitis and the number of missing teeth in subjects diagnosed as having missing teeth, a simple comparison cannot be made. However, ORs of the group “28 missing teeth” were higher than those of the group “1–9 teeth present” (Tables 3 and 4). Namely, ORs in subjects diagnosed as having missing teeth were greater than those in subjects with periodontitis. The same trend was observed when aspiration pneumonia was used as an outcome variable in logistic regression models adjusting for sex and age group [22]. The difference in ORs might be ascribed to the difference in the number of teeth present between study subjects diagnosed as having missing teeth and those diagnosed with periodontitis. For example, edentulous subjects, who have a higher risk of Alzheimer’s disease than dentulous subjects [8, 15], were included in the group of patients diagnosed as having missing teeth, but not in the group of patients diagnosed with periodontitis.

To the best of our knowledge, this is the first study to describe the prevalence of Alzheimer’s disease in patients visiting dental offices. The prevalence in patients in their 60s varied 0.2%–0.4% and rose with age to 12%–16% in patients aged 85 years or older. The national survey in Japan reported the mean prevalence of dementia in people aged ≥65 years to be 15.8% (95% CI: 12.4–22.2) [32] and Alzheimer’s disease was confirmed as the predominant type of dementia, accounting for 65.8% of all cases. The estimated percentage of Alzheimer’s disease from the data (10.4%) is higher than the prevalence of Alzheimer’s disease in the present study (3.0% and 3.7% in subjects diagnosed as having periodontitis and missing teeth, respectively). The low prevalence of Alzheimer’s disease in this study cohort might be attributed to several factors, including difficulties in providing dental treatment to patients with severe cognitive impairment and lack of subjective symptoms of oral diseases in patients with severe cognitive impairment [26]. The Japanese government launched a new national dementia strategy to tackle the increasing number of people with dementia. The goal is to strive toward a society in which people can live with a positive outlook even if they have dementia. Roles of dentists in this strategy include early detection of dementia through routine dental checkups and collaboration with medical doctors to support patients with dementia from the viewpoint of maintaining and improving oral health. The results of the present study suggest increased future needs of oral health promotion and care of people with Alzheimer’s disease.

Although the primary strength of the present study was its large sample size, which covered 9.6% and 1.6% of all older Japanese subjects diagnosed with periodontitis and missing teeth, respectively, there are several limitations. First, the study cohort comprised patients visiting dentists, but not those with healthy teeth and periodontium. Therefore, the results from the present study cannot be generalized to older people in Japan. For example, subjects with moderate or serious Alzheimer’s disease might not visit dentists. Second, the numbers of teeth present and missing teeth were calculated from dental formulas. In particular, the number of missing teeth includes abutment teeth for bridges because the dental formula for bridges includes both missing and abutment teeth. Therefore, the number of missing teeth for a relatively small number of missing teeth may be overestimated. However, we categorized the number of missing teeth into three groups, 1–13, 14–27, and 28, to minimize this limitation. Third, the severity of periodontitis is unknown because this information was not included in the NDB. Finally, possible confounders, including socioeconomic factors [33], were not included in logistic regression models because this information was not included in the NDB.

## Conclusions

This cross-sectional study using data of people aged 60 years or older diagnosed as having periodontitis or missing teeth from the National Database of Health Insurance Claims and Specific Health Checkups of Japan showed that patients with fewer teeth present and a greater number of missing teeth had a higher risk of Alzheimer’s disease after adjusting for sex and age.
